# Calibrating ultrasonic sensor measurements of crop canopy heights: a case study of maize and wheat

**DOI:** 10.3389/fpls.2024.1354359

**Published:** 2024-06-05

**Authors:** Yudong Zheng, Xin Hui, Dongyu Cai, Muhammad Rizwan Shoukat, Yunling Wang, Zhongwei Wang, Feng Ma, Haijun Yan

**Affiliations:** ^1^ College of Water Resources and Civil Engineering, China Agricultural University, Beijing, China; ^2^ Institute of Dryland Farming, Hebei Academy of Agriculture and Forestry Sciences, Key Laboratory of Crop Drought Resistance Research of Hebei Province, Hengshui, Hebei, China; ^3^ College of Resources and Environmental Sciences, China Agricultural University, Beijing, China; ^4^ Hebei Science and Technology Innovation Service Center, Shijiazhuang, Hebei, China; ^5^ College of Animal Science and Technology, Hebei Agricultural University, Baoding, Hebei, China; ^6^ State Key Laboratory of Efficient Utilization of Agricultural Water Resources, China Agricultural University, Beijing, China

**Keywords:** ultrasonic sensor, canopy height, maize, wheat, normalized difference vegetative index, calibration mode

## Abstract

Canopy height serves as an important dynamic indicator of crop growth in the decision-making process of field management. Compared with other commonly used canopy height measurement techniques, ultrasonic sensors are inexpensive and can be exposed in fields for long periods of time to obtain easy-to-process data. However, the acoustic wave characteristics and crop canopy structure affect the measurement accuracy. To improve the ultrasonic sensor measurement accuracy, a four-year (2018−2021) field experiment was conducted on maize and wheat, and a measurement platform was developed. A series of single-factor experiments were conducted to investigate the significant factors affecting measurements, including the observation angle (0−60°), observation height (0.5−2.5 m), observation period (8:00−18:00), platform moving speed with respect to the crop (0−2.0 m min^−1^), planting density (0.2−1 time of standard planting density), and growth stage (maize from three−leaf to harvest period and wheat from regreening to maturity period). The results indicated that both the observation angle and planting density significantly affected the results of ultrasonic measurements (p-value< 0.05), whereas the effects of other factors on measurement accuracy were negligible (p-value > 0.05). Moreover, a double-input factor calibration model was constructed to assess canopy height under different years by utilizing the normalized difference vegetation index and ultrasonic measurements. The model was developed by employing the least-squares method, and ultrasonic measurement accuracy was significantly improved when integrating the measured value of canopy heights and the normalized difference vegetation index (NDVI). The maize measurement accuracy had a root mean squared error (RMSE) ranging from 81.4 mm to 93.6 mm, while the wheat measurement accuracy had an RMSE from 37.1 mm to 47.2 mm. The research results effectively combine stable and low-cost commercial sensors with ground-based agricultural machinery platforms, enabling efficient and non-destructive acquisition of crop height information.

## 1 Introduction

Crop height is an essential agriculture parameter (Yuan et al., 2018) closely related to yield (Bablu and Ritchie, 2014), above-ground biomass (Pittman et al., 2015), and lodging (Singh et al., 2019). The measurement of crop height in traditional agronomic practices primarily relies on manual methods, which has problems such as random deviations, large time consumption, and low efficiency. Ultrasonic sensors can be used to obtain crop height information through non-contact methods (Barmeier et al., 2016). Ultrasonic sensors’ data is easy to process, and this type of sensor is distinguished by its cost-effectiveness, ease of portable installation (Jeon et al., 2011), and suitability for prolonged exposure in field environments, particularly when compared with light detection and ranging (LiDAR) (Yuan et al., 2019; Dou et al., 2021) and unmanned aircraft system (UAS) imagery (Gai et al., 2015; Jiang et al., 2016).

Ultrasonic sensors are widely used in the field of distance measurement, and this type of sensor was used in phenotyping studies on maize (Li et al., 2020; Aziz et al., 2004), wheat (Pittman et al., 2015; Scotford and Miller, 2016), rice (Shibayama et al., 1985), sorghum (Shibayama et al., 1985), and soybean (Pittman et al., 2015). However, ultrasonic sensor measurements have been found to underestimate canopy height in many studies (Fricke et al., 2011; Yuan et al., 2018; Sui and Baggard, 2018). In related studies, using ultrasonic sensors to measure canopy height, the RMSE was 13−340 mm. Chang et al. (2017) and Farooque et al. (2013) found that the ultrasonic measurement errors of blueberry canopy height were in the range of 13−57 mm. The measurement error of soybean canopy height was about 30 mm in the ultrasonic experiment conducted by Kataoka et al. (2002). Sui and Baggard (2018) found that the ultrasonic measurement errors of soybean or cotton canopy heights were in the range of 31−58 mm. Yuan (2019) obtained wheat canopy height using static ultrasonic measurements with an RMSE of 340 mm. Aziz et al. (2004) compared ultrasonic measurements with manual measurements of maize canopy height, and found the fitted regression line with R^2^ of 0.41. Combining the results of previous studies with the field measurements in this study, the measurement error of wheat and maize (the RMSE of 340−976mm) is larger than that of other crops. Considering that maize and wheat are major food crops, it makes a lot of sense to conduct research on them. Thus, it is necessary to study the ultrasonic measurements accuracy on canopy to improve the use of ultrasonic sensors in agricultural management systems.

The measurement of canopy geometry using ultrasonic sensors is based on the time-of-flight method. This type of sensor emits acoustic pulse signals during operation and forms reflected echoes on the reflective surface of the measured object (Manual-Senix-TSPC-Family, 2020). In some measurement situations, when the reflective surface has an irregular shape and a large surface roughness, it interferes with the formation of reflected echoes, ultimately affecting the ranging results (Ma and Shen, 1983; Jeon et al., 2011). Owing to the dense-gap structure and irregular reflective surface of the plant canopy (Shibayama et al., 1985; Mckerrow and Harper, 2002; Nan et al., 2019), the ultrasonic measurements of observation targets such as grasslands, shrubs, and forests may deviate from the actual canopy height (Sui and Baggard, 2018; Aziz et al., 2004; Nan et al., 2019). Based on the principle of sound wave propagation, ultrasonic waves have directionality (Feng, 1999). Meanwhile, when the measurement target is too close to the sensor, the successive emitted ultrasonic waves can interfere with the reception of the signal. Thus, ultrasonic sensors have measurement blind spots (Pan, 2006). Moreover, when ultrasonic waves propagate in various media, they are attenuated via scattering, absorption, and diffusion. When there is relative motion between the sound source and the observation target, a Doppler frequency shift also occurs (Feng, 1999), and the speed of sound changes owing to varying air pressure, temperature, and humidity. Therefore, many factors affect the measurement accuracy of ultrasonic sensors. However, it is unknown which factors can significantly interfere with the actual measurement results in the field. To date, some researchers have conducted relevant ultrasonic experiments on canopy measurements. Andújar et al. (2012) used ultrasonic sensors for the detection of weeds in cereal crops and concluded that the density of plants may influence the reflected intensity of ultrasound. Barmeier et al. (2016) measured the height of barley using ultrasonic sensors and found that high speed movement during measurement, leaf angle, leaf size and canopy coverage area may have affected the measurements. Llorens et al. (2011) found the well correlation between ultrasonic measurements and leaf area index. Nan et al. (2019) found little difference in the relative error distribution at different distances (0.8−1.2 m) Shibayama et al. (1985) and Fricke et al. (2011) found that the ultrasonic reflection signal was highly correlated with canopy leaf inclination angle, blade area, and leaf density. At the same time, considering that vegetation index such as normalized difference vegetation index (NDVI) can provide a more intuitive status of canopy cover (Fawcett et al., 2020) to researchers, many scholars have tried to combine spectral reflectance sensors with distance measurement devices that can be used for monitoring canopy physical characteristics such as canopy height (Schirrmann et al., 2017; Singh et al., 2019), above ground biomass (Liu et al., 2022; Yue et al., 2017), and leaf area index (Bian et al., 2023; Liu et al., 2023). Few studies have focused on identifying the major factors affecting ultrasonic measurement accuracy through field experiments or on proposing specific calibration methods for improving the accuracy of ultrasonic measurement.

Therefore, it is necessary to conduct ultrasonic sensor experiments on the canopy of crop populations in the field to determine the factors (such as environmental factors, sensor measurement methods, crop growth stages and planting status) that influence the measurements. In addition, because the environmental conditions of field experiments are not entirely controllable, a single-factor experiment can be performed to determine the factors that significantly affect the ultrasonic measurement results. Then an ultrasonic measurement calibration model can be established based on these influencing factors. Hence, research objectives were to (1) construct a system to acquire canopy information(ultrasonic and NDVI data) stably and efficiently based on a center pivot, (2) determine the main factors affecting the measurement of canopy height by ultrasonic sensors through multi-year field experiments on maize and wheat, and (3) establish a calibration model to improve the accuracy of ultrasonic measurements.

## 2 Materials and methods

### 2.1 Setting up the canopy height measurement platform

**Figure 1:**
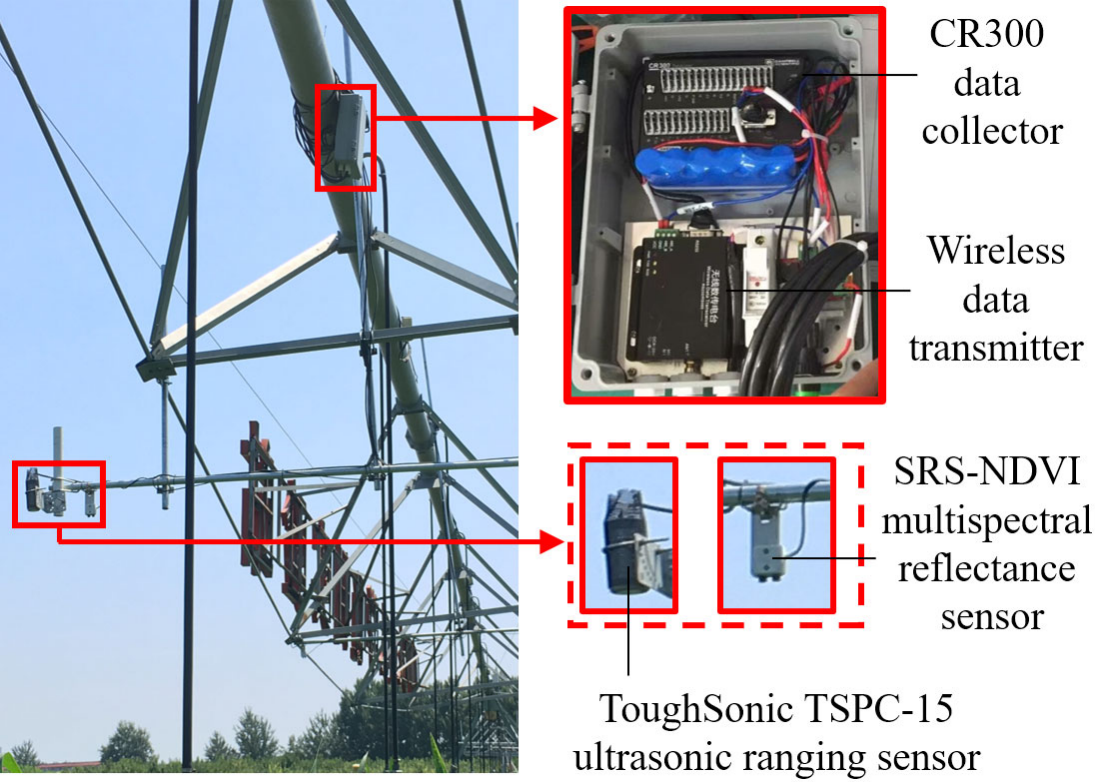
Experimental setup and sensor configuration.

A canopy height measurement system was installed on a center pivot (**Figure 1**) in this study. The main components were integrated into a waterproof control cabinet, including an ultrasonic sensor (ToughSonic TSPC−15, Senix Corporation, Inc. USA), a multispectral reflectance sensor (SRS−NDVI, METER Group, Inc. USA), and a data logger (CR300, Campbell Scientific, Inc. USA). The ultrasonic sensors have a Field of View (FoV) of 14°and a measurement range of 0.25−9.1 m (accuracy is better than 0.5% of target distance). The ultrasonic transmission frequency is 75 kHz. The ultrasonic sensor used in this experiment had a rotating base (**Figure 1**) to adjust observation direction. The multispectral reflectance sensor (measurement bands of red in the range 640−660 nm and near-infrared in the range 800−820 nm) consisted of up-looking and down-looking probes. The up-looking probe was pointed vertically to the sky without any shading object and had a field of view of 180° to receive radiation from the sky. The down-looking probe pointing (**Figure 1**) was vertical to the canopy and had a FoV of 36° to receive radiation from the canopy. The multispectral reflectance sensor acquired observational data once per second and sent the average value to the laptop every 15 s during the measurement.

### 2.2 Experiment factor determination and field measurements

#### 2.2.1 Experiment site and crop cultivation

The experiment was conducted at the Tongzhou Experimental Station of China Agricultural University (Beijing, China, 39°41’59” N, 116°41’01” E) from 2018 to 2021. The organic matter content in the 0−40 cm layer was 12.3 g kg^−1^ and the soil type was sandy loam. The main physicochemical properties of root zone soil (0−100 cm) such as bulk density was 1.5 g cm^−3^, ammonium nitrogen content was 3.4 mg kg^−1^, nitrate nitrogen content was 10.1 mg kg^−1^, available phosphorus was 26.2 mg kg^−1^, available potassium was 149.8 mg kg^−1^, and pH was 8.3.

The maize variety used was Nongda 86 and the wheat variety was Nongda 211. The maize seeding rate was set at 37.5 kg ha^−1^ with a row spacing of 60 cm and plant spacing of 20 cm. The wheat seeding rate was 277.5 kg ha^−1^ with a row spacing of 15 cm. Both maize and wheat were fertilized uniformly based on local fertilizer management. For maize, the total amounts of nitrogen, phosphorus (P_2_O_5_), and potassium (K_2_O) applied were 225, 105, and 95 kg ha^−1^, respectively. All phosphorus and potassium fertilizers and 45 kg ha^−1^ of nitrogen were applied as basal fertilizers. The remaining nitrogen was applied at the sixth-leaf stage (72 kg ha^−1^) and twelve-leaf stage (108 kg ha^−1^). For wheat, the total amounts of nitrogen, phosphorus (P_2_O_5_), and potassium (K_2_O) applied were 278, 150, and 90 kg ha^−1^, respectively. All phosphorus and potassium fertilizers and 68 kg ha^−1^ of nitrogen were applied as basal fertilizers. The remaining nitrogen was applied at the regreening (84 kg ha^−1^), jointing (84 kg ha^−1^) and filling stages (42 kg ha^−1^). Plant growth in maize (Siebers et al., 2017) and wheat (Livingston et al., 2016) was categorized into vegetative and reproductive stages. In this experiment, the maize (harvested as silage) growth stages were divided into three categories: early vegetative (V3−V6), late vegetative (V7−V12), and reproductive (VT−R). The wheat growth stages were subdivided into vegetative (regreening to jointing), reproductive (heading to early filling), and maturation (late filling to maturity) stages. Time division of each growth stage are shown in the **Table 1**.

**Table 1:** Time division of each important growth stage of maize and wheat in experiment.

	Experiment year	Early vegetative	Post vegetative	Reproductive
		VE–V6 (DD/MM)	V7–V14 (DD/MM)	VT–R (DD/MM)
Maize	2018	07/07–03/08	04/08–18/08	19/08–30/09
	2019	02/07–30/07	31/07–14/08	15/08–29/09
	2020	04/07–01/08	02/08–19/08	20/08–05/10
	Experiment year	Vegetative	Reproductive	Maturation
		Regreening –Jointing (DD/MM)	Heading –Early-filling (DD/MM)	Late-filling–Maturity (DD/MM)
Wheat	2019	19/03–30/04	01/05–14/05	15/05–20/06
	2020	14/03–01/05	02/05–13/05	14/05–17/06
	2021	16/03–03/05	04/05–16/05	17/05–18/06

VE is emergence stage, V6 is sixth-leaf stage, V7 is seventh-leaf stage, V14 is fourteen-leaf stage, VT is tasseling stage, and R is harvesting stage.

#### 2.2.2 Field measurements of the maize and wheat canopy

The requirement for the canopy height measurement (Liu et al., 2023) is obtaining the natural height of the first fully expanded leaf (before maize/wheat heading) or the ear excluding the awn (after maize/wheat heading). The calculation method for ultrasonic measurement (Ma and Shen, 1983) of canopy height is as follows:


(1) $$\text{CH}=\text{L}-\frac{1}{2}\text{c}\times {\text{t}}_{\text{r}}$$

(2) $$\text{c}=\sqrt{\frac{{\text{γP}}_{0}{\text{T}}_{\text{air}}}{0.00348{\text{P}}_{0}-0.00134{\text{h}}_{\text{r}}{\text{ P}}_{\text{sb}}}}$$

where CH is the canopy height (mm), L is the vertical distance from the ultrasonic sensor to the ground surface (mm), c is the propagation speed of ultrasonic waves in the air medium (m s^−1^), t_r_ is the time interval from sending signal of the ultrasonic sensor to receiving reflected signal of observed canopy(s), and γ is the air constant. Where P_0_ is the atmospheric pressure (Pa), T_air_ is the air temperature (°C), h_r_ is the air relative humidity (%), and P_sb_ is the saturated water vapor partial pressure (Pa).

The measurement accuracy of ultrasonic sensors is affected by several factors (Jeon et al., 2011; Nan et al., 2019) during the field experiments. As shown in Equation (1), sound speed and ultrasonic reflection time are the main factors affecting measurement accuracy. The ultrasonic wave has directionality and attenuates during propagation in air. Furthermore, a Doppler shift occurs when there is relative motion between the sound source and the observed target. Hence, the sensor’s observation angle (angle between the sensor observation direction and the vertical downward direction), observation height (distance from the sensor to target canopy in observation direction), and moving speed (the sensor moving speed with respect to target canopy) have the potential to be factors that significantly affect the measurement results. The factors affecting the speed of sound in Equation (2) are atmospheric pressure, temperature, and humidity in the plain area under normal air conditions. The overall terrain of cropland in the North China Plain is flat and the atmospheric pressure remains stable, making it challenging to adjust. Hence, atmospheric pressure was not considered in this study. In addition, the changes in temperature and humidity of the field environment can be controlled by observation periods (the measuring time during a day). Moreover, changes in canopy coverage (planting density and growth stage) may affect the continuity of its acoustic wave reflection surface. The experiment was conducted under no-wind conditions to avoid wind interference with the canopy height and airflow during ultrasonic propagation.

Based on above assumptions, this study conducted a single-factor experiment to filter out the significant influencing factors that cause deviations. The specific combinations and values of experimental parameters are presented in **Table 2**. In this table, observation angle ( θ ) as selected at 0°, 15°, 30°, 45°, and 60° in the range of 0−60°, and the canopy heights were calculated from the raw data obtained at different observation angles using the formula by $\text{CH}=\text{L}-\frac{1}{2}\text{c}\times {\text{t}}_{\text{r}}\times \text{cosθ}$ . The planting density (D) used the normal seeding rate (277.5 kg ha^−1^ of wheat, 37.5 kg ha^−1^ of maize) as the standard planting density (d). The values of d ranged from 0.2 d to 1.0 d, increasing in increments of 0.2d and specifically including 0.2 d, 0.4 d, 0.6 d, 0.8 d, and 1.0 d. The observation heights (H) were selected at intervals of 0.5 m within the range of 0.5 m to 2.5 m, specifically at 0.5 m, 1.0 m, 1.5 m, 2.0 m, and 2.5 m. The observation periods (t) were selected from the time intervals of 8:00–10:00, 10:00–12:00, 12:00–14:00, 14:00–16:00, and 16:00–18:00. The moving speeds (v) were selected as 0, 0.5, 1.0, 1.5, and 2.0 m s^–1^, within the range of 0–2.0 m min ^–1^.

**Table 2:** Single-factor experimental parameters used in this study.

Parameters	θ (°)	D	H (m)	t	v (m min^–1^)
θ	0–60	d	1.0	12:00–14:00	2
D	0	(0.2–1.0) d	1.0	12:00–14:00	2
H	0	d	0.5–2.5	12:00–14:00	2
t	0	d	1.0	8:00–18:00	2
v	0	d	1.0	12:00–14:00	0–2

θ (observation angle) is 0°, 15°, 30°, 45°, and 60°, D (planting density) is 1.0d (d, standard planting density: the wheat seeding rate is 277.5 kg ha^–1^, the maize seeding rate is 37.5 kg ha^–1^), 0.8d, 0.6d, 0.4d, and 0.2d, H (observation height) is 0.5, 1.0, 1.5, 2.0, 2.5 m, t (observation period) is selected from 8:00–10:00, 10:00–12:00, 12:00–14:00, 14:00–16:00, and 16:00–18:00, and v (moving speed) is 0, 0.5, 1.0, 1.5, and 2.0 m min^–1^.

A series of data were measured for a more comprehensive description of canopy cover status. Data types include the total area of green leaves per unit land surface area (leaf area index, LAI), the normalized difference vegetation index (NDVI), the measured value of the canopy height by ultrasonic sensor (CH_m_) and the actual value of the canopy height (CH_a_). The sampling date is listed in **Table 3**.

**Table 3:** Sampling dates of each growth stage in maize and wheat fields.

	Sampling year	Sampling date (DD/MM)		
		Early vegetative	Post vegetative	Reproductive
Maize	2018	01/08	08/08, 14/08	19/08, 08/09, 21/09
	2019	11/07, 17/07, 28/07	31/07, 04/08	15/08
	2020	18/07, 30/07	08/08, 15/08	20/08
	Sampling year	Sampling date		
		Vegetative	Reproductive	Maturation
Wheat	2019	04/04, 20/04	03/05, 14/05	25/05
	2020	19/04	12/05	22/05
	2021	07/04, 21/04	14/05	27/05

LAI was calculated from crop images using a canopy image analysis software (Guanceng Fenxi.V1.0, Fansheng Technology. China), and the canopy images of crops under clear and cloudless skies without intense sun exposure at different growth stages were obtained using a canopy image analyzer (DC−2000, Fansheng Technology. China). This canopy image analyzer is similar to the LAI2200, and DC−2000 analyzer calculates LAI from light measurements made with a “fish-eye” optical sensor (148° of FoV). Measurements made above and below the canopy were used to calculate canopy light interception at five zenith angles, from which LAI was computed using a model of radiative transfer in vegetative canopies. The measurement position was selected within 2 m×2 m of the measuring area of the ultrasonic sensor. Following the instruction manual for row crops, ground measurements were made along diagonal transects between the rows (Fang et al., 2014). Two repeats were conducted for each measurement, with one reading taken above the canopy and six readings taken (uniform distribution) below the canopy. For below canopy measurements, ensure that the distance between the leaves and the “fish−eye” optical sensor was at least 4 times the maximum width of the leaves. All LAI values obtained in the 2 m×2 m area were averaged to obtain the LAI value for each measurement position.

NDVI is relatively mature and is widely used to observe plant canopies (Tucker, 1979). The calculation Equations (3–5) formula is as follows:


(3) $$\text{NDVI}=\frac{{\text{R}}_{\text{NIR}}-{\text{R}}_{\text{RED}}}{{\text{R}}_{\text{NIR}}+{\text{R}}_{\text{RED}}}$$

(4) $${\text{R}}_{\text{NIR}}=\frac{{\text{r}}_{\text{down}-\text{NIR}}}{{\text{r}}_{\text{up}-\text{NIR}}}$$

(5) $${\text{R}}_{\text{RED}}=\frac{{\text{r}}_{\text{down}-\text{RED}}}{{\text{r}}_{\text{up}-\text{RED}}}$$

where R_NIR_ is the reflectivity data in the near-infrared band, R_RED_ is the reflectivity data in the red band, r_down-NIR_ is the radiation intensities of the canopy in the near-infrared band, r_down-RED_ is the radiation intensities of the canopy in the red bands, r_up-NIR_ is the radiation intensities of the sky in the near-infrared, and r_up-RED_ is the radiation intensities of the sky in the red bands.

CH_m_ and CH_a_ were calculated for canopy height measurements as follows Equations (6, 7):


(6) $${\text{CH}}_{\text{m}}=\text{L}-{\text{l}}_{\text{MV}}$$

(7) $${\text{CH}}_{\text{a}}=\text{L}-{\text{l}}_{\text{AV}}$$

where L is the total distance from the ultrasonic sensor to the soil surface below the canopy, ${\text{l}}_{\text{MV}}$ the distance from the canopy surface measured by the ultrasonic sensor, and ${\text{l}}_{\text{AV}}$ the manually measured distance between the ultrasonic sensor and the canopy surface. Additionally, engineering tape (Autlock 5 m, Bosch of Shanghai, Inc. China) was used for manual measurements.

The transplanted canopy and in-situ canopy were used as different observation platforms to meet the requirements of parameter changes in different single-factor experiments.

(1) Measurement with transplanted canopy.

**Figure 2:**
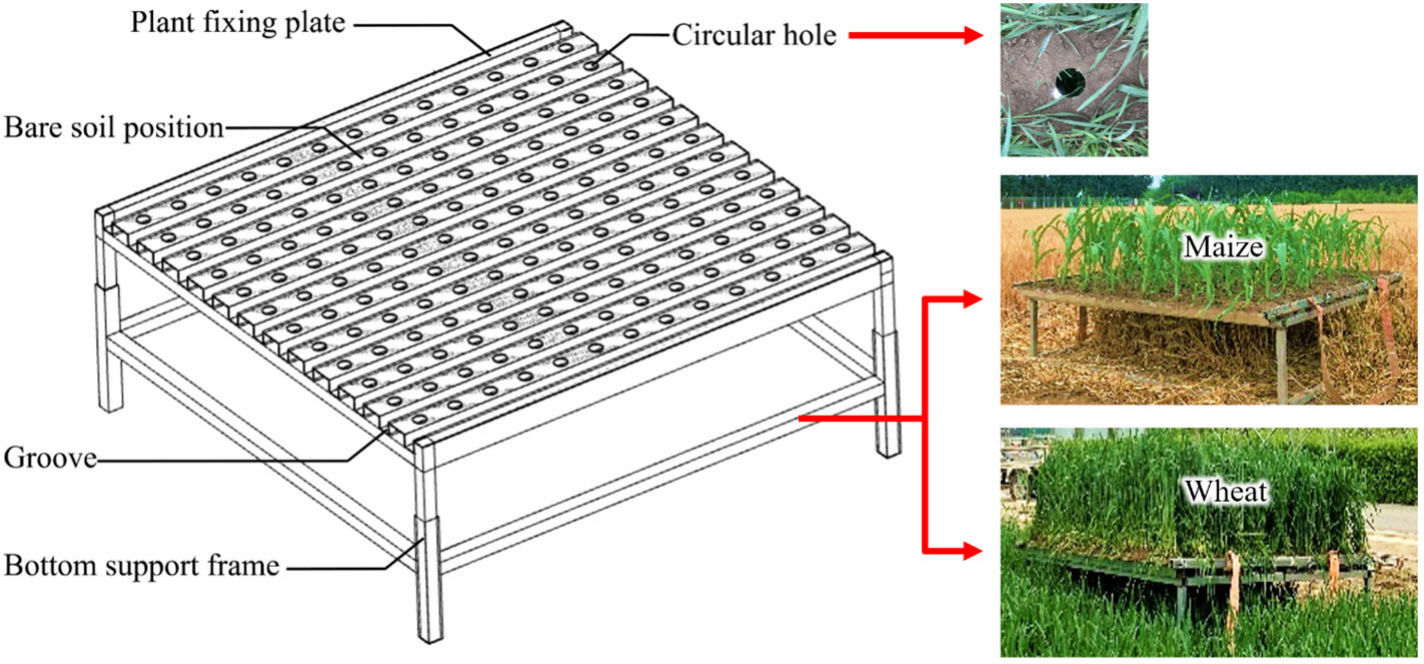
Schematic diagram and scene photographs of the crop fixing platform.

Crop density needs to be flexibly changed to perform single-factor experiments on planting density. Therefore, a crop-fixing platform was constructed and the experiment was conducted in 2019. The structural composition of this platform included a plant fixing plate with an area of 2 m × 2 m and a bottom support frame (**Figure 2**). The fixed plate had grooves determined according to the spacing of the crop rows, and these grooves were used for transplanting the root systems of the crop plants. The number of fixed crop plants in the groove was determined by the planting density. A thin layer of soil was covered over the surface of the fixed plate to restore the actual observation environment after the canopy was transplanted. In addition, to ensure the freshness and stable shape of the transplanted crop plants during the observation period, the experiment was conducted under weak sunlight conditions, and the soil around the crop roots was regularly watered every half hour. A circular hole with a diameter of 8 cm was formed in the bare soil position between the grooves of the fixed plate to facilitate the fish-eye lens to observe the canopy from the bottom of the plant upward. Single-factor experiments at different viewing angles were also conducted using this platform. Three sets of canopy fixed plates at the same growth stage were measured separately as replicates of the observation experiment on the transplanted canopy. For each replicate, three manual measurement points were uniformly and randomly selected on the fixed platform to obtain the CH_a_, and the ultrasonic sensor was used to continuously observe each manual measurement point for 3 min to obtain the CH_m_. In addition, both CH_a_ and CH_m_ were calculated by averaging the measurement point data.

Ultrasonic sensors were initially designed to measure continuous solid and liquid surfaces in the industrial sector (Manual-Senix-TSPC-Family, 2020). To verify the original measurement reliability of this type of sensor, the crop canopy was replaced with a bare soil slab (referred to as “bare-soil slab”) for height measurement under each experimental condition.

(2) Measurement with in-situ canopy.

**Figure 3:**
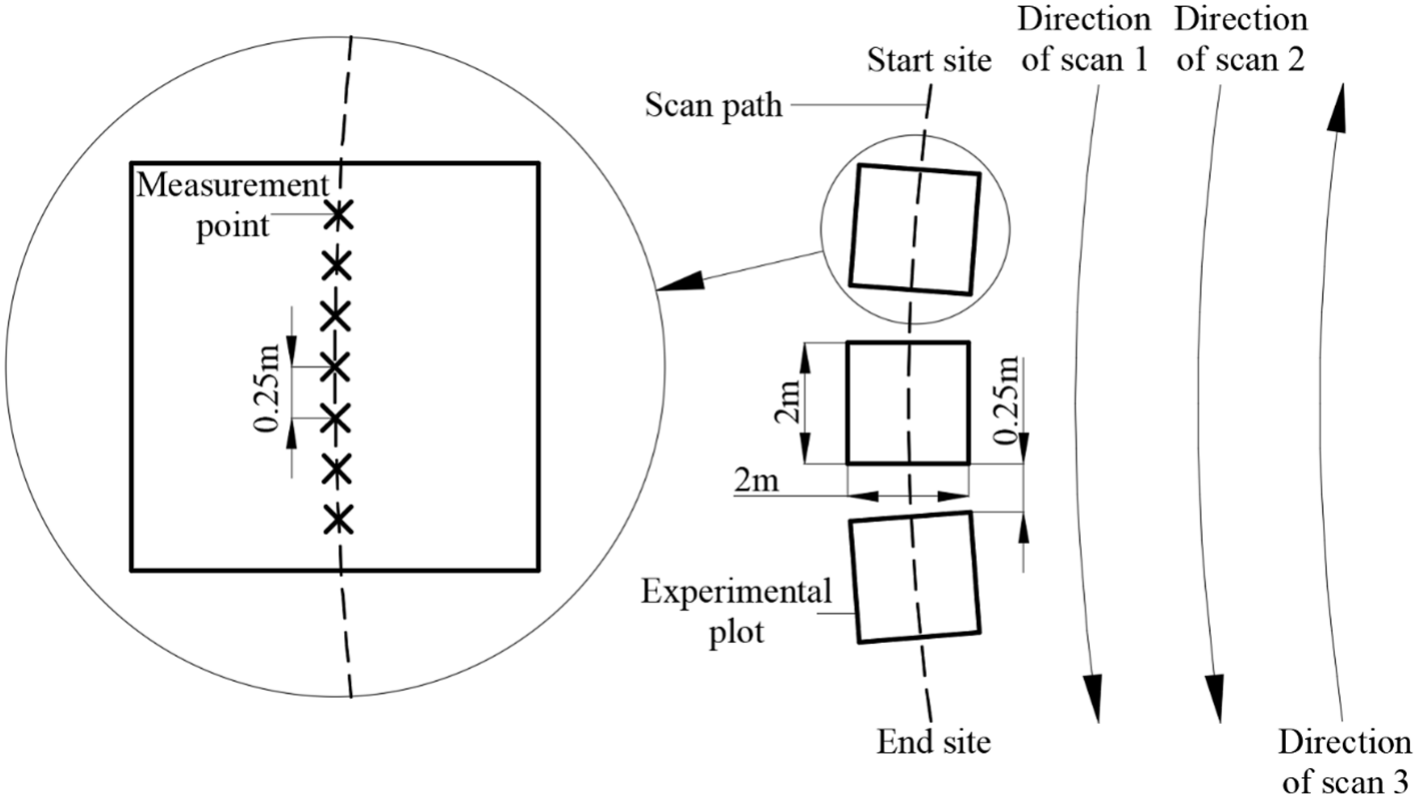
Schematic diagram of the in-situ canopy observation experiment.

**Figure 3** shows that the field in each treatment was divided into three experimental plots of approximately 2 m × 2 m located on the scan path of the measurement system. A single-factor experiment was performed for H, t, T, and v in 2019. The spacing between adjacent experimental plots is 0.5−1.0 m. A uniform distribution of three to seven measurement points was selected randomly in each plot during the experiment. The data acquisition methods for CH_a_ and CH_m_ were the same as those used in the transplanted canopy observation experiment.

In the single-factor experiment of v, the system completed CH_m_ acquisition from the start site to the end site at a specific moving speed (**Figure 3**). Manual measurements were taken every 25 cm on the scan path, and the average value was taken as the value of CH_a_. Moreover, three scans (Scans 1, Scans 2, and Scans 3) were conducted for each case to study the repeatability (Walter et al., 2019) of the ultrasonic measurement. The measurements of Scans 1 and Scans 2 were used to compare the repeatability in the same direction, and the measurements of Scans 1 and Scans 3 were used to compare the repeatability in the opposite direction.

In addition, field measurements were carried out on maize (2018, 2019, and 2020) and wheat (2019, 2020, and 2021) for several years to investigate the general applicability of the ultrasonic system for in-situ canopy height.

### 2.3 Experimental data analysis

The Pearson correlation coefficient (r) between the relative error (percentage of the absolute error value over the true value) and the experimental parameters was calculated using the SPSS software (IBM Corp., Armonk, NY, USA) to explore the correlation between changes in the single environmental factor and measurements. Analysis of variance (ANOVA) was used in the same software to evaluate the effects of single-factor experimental parameters on the ultrasonic measurements. In addition, before performing ANOVA, the normality of the samples was verified using the Shapiro-Wilk normality test, and the variance homogeneity of the samples was verified using the Levene test.

In this study, a CH_m_ calibration model was established using the multiple regression method in Origin 8.5 software (OriginLab, Northampton, MA, USA), and the output value of the calibration model was the calibration value of canopy height (CH_c_). The coefficient of determination R^2^, and root mean square error (RMSE) were used for model evaluation by Equations (9, 10). The variance inflation factor (VIF) was chosen as the indicator of multicollinearity to avoid variables with severe multicollinearity from distorting the estimation of the regression model (Doi, 2019). It is calculated as follows in Equation 8:


(8) $$\text{VIF}=\frac{1}{1-R_{\text{i}}^{2}}$$

where $R_{\text{i}}^{2}$ is based on the variance of the ith independent variable around its mean that is explained by the other independent variables in the model. When the VIF > 10 (Miles, 2014), it indicates that the collinearity between the variables is too strong, so that the model cannot be reasonably constructed using these variables.


(9) $${\text{R}}^{2}=1-\frac{\sum_{\text{i}=1}^{\text{n}}{\left({\text{CH}}_{\text{c i}}-{\text{CH}}_{\text{a i}}\right)}^{2}}{\sum_{\text{i}=1}^{\text{n}}{\left({\text{CH}}_{\text{c i}}-{\bar{\text{CH}}}_{\text{a}}\right)}^{2}}$$

(10) $$\text{RMSE}=\sqrt{\frac{\sum_{\text{i}=1}^{\text{n}}{\left({\text{CH}}_{\text{c i}}-{\text{CH}}_{\text{a i}}\right)}^{2}}{\text{n}}}$$

In the above formula: ${\bar{\text{CH}}}_{\text{a}}=\frac{1}{\text{n}}(\sum_{\text{i}=1}^{\text{n}}{\text{CH}}_{\text{a i}})$ , CH_ci_ is the value of the i-th CH_c_ data, ${\text{CH}}_{\text{a i}}$ is the value of the i-th CH_a_ data, n is the number of samples.

## 3 Results and analysis

### 3.1 Original measurement reliability and repeatability of ultrasonic sensor

It was necessary to verify the original measurement reliability of the ultrasonic sensor before conducting the crop canopy experiment. The results (**Supplementary Figure S1**) show that the correlation between the measured values and the actual height of the bare-soil slab had R^2^ values larger than 0.99 because a bare and flat soil surface has a much better smoothness than a crop canopy surface. And the average relative error of measurements was less than 0.51%. The above data indicates that the ultrasonic sensor had good measurement reliability.

**Supplementary Figure S2** shows high repeatability of the ultrasonic sensor during the data collection period for measurement results. Data collection movements in the same and opposite directions showed no significant differences in the measurements. The R^2^ values were higher than 0.97, the slopes were close to 1, and the intercept values for all measured fits were below 5 mm.

### 3.2 Correlation between the actual and measured values of canopy height

**Figure 4:**
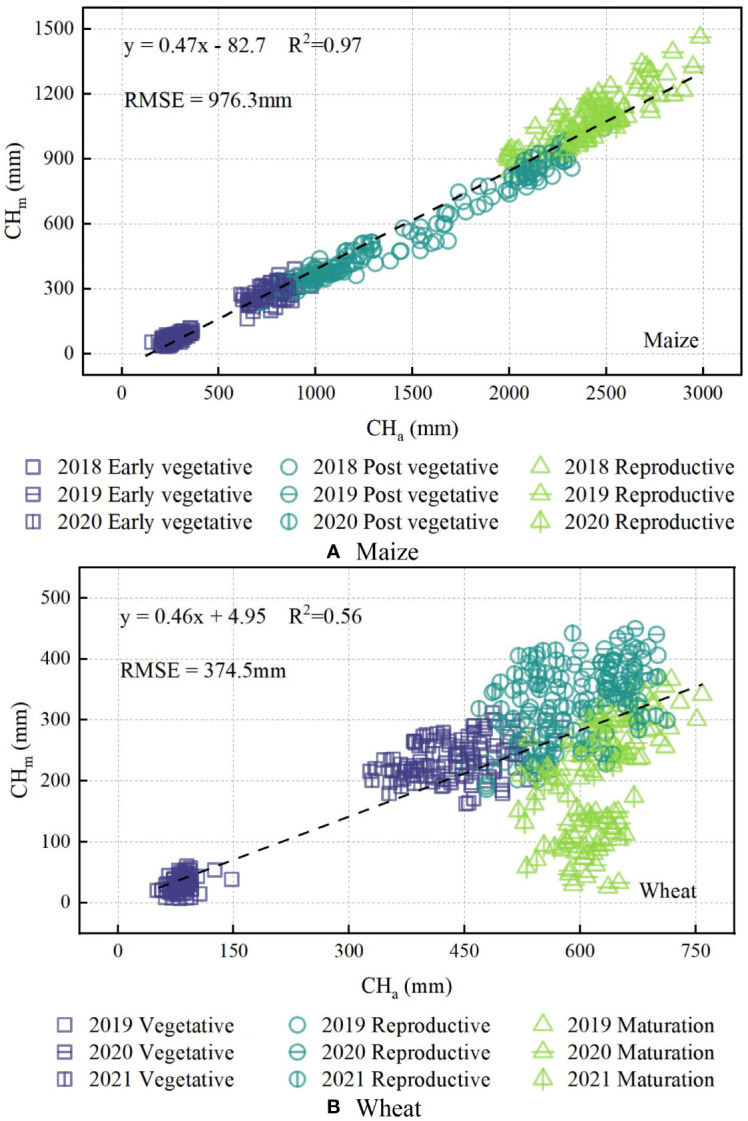
Relationship between the actual value of canopy height (CH_a_) and the measured value of canopy height (CH_m_) by ultrasonic sensor during all sampling growth stages from 2018 to 2021 of maize **(A)** and wheat **(B)**. The dashed lines indicate the best fit line.

**Figure 4** shows that maize had significantly better linear fitting results for all growth stages than that of wheat. This is because the maize plant leaves had not yet wilted and shed in large numbers during the silage harvesting stage. Moreover, CH_m_ (33.2−1462.1 mm for maize, 51.4−758.0 mm for wheat) and CH_a_ (155.5−2984.3 mm for maize, and 7.4−450.7 mm for wheat) showed consistency to some extent. However, the value obtained using the ultrasonic sensor was much lower than the actual value (measurement accuracy RMSE=374.5−976.3 mm) and was not suitable for direct use. Therefore, further experiments are required to determine the influencing factors, and the measurement results must be calibrated.

### 3.3 Experiment parameter correlation analysis

**Figure 5:**
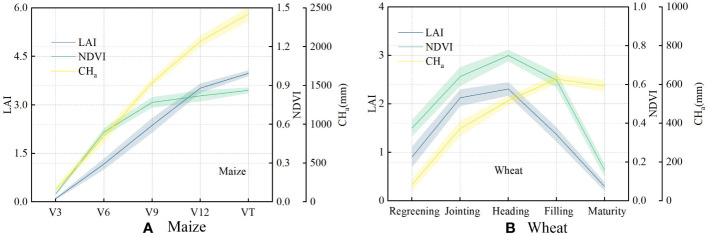
Variation in trends of the leaf area index (LAI), normalized difference vegetation index (NDVI), and actual value of canopy height (**CH_a_**) in each observed crop growth stage of maize **(A)** and wheat **(B)**.

The changing trends in the main canopy parameters with the growth and development of canopies are shown in **Figure 5**. V3−VT is part of the vegetative growth stage of maize. The canopy coverage increased during these stages as the leaves grew. The LAI increased from 0.103 to 3.979, and the NDVI increased from 0.062 to 0.863. In addition, the canopy height of the maize plants increased from 161 to 2421 mm as the plants grew. For wheat, the greening to heading stages are part of the vegetative growth stage, and there was a similar trend for the change in each parameter, as for maize. Wheat canopy height (from 83 to 628 mm), LAI (from 0.905 to 2.307), and NDVI (from 0.375 to 0.821) show the gradually increasing trend. The wheat heading to maturity stages belong to the reproductive growth stage, and the wheat height stops growing and leaves begin to fade during this time. There was no change in the wheat canopy height, whereas the LAI decreased to 0.291, and the NDVI decreased to 0.158.

The canopy structures during the maize VT and wheat heading stage were relatively complete and stable. Therefore, variables such as θ, D, H, t, and v were analyzed during these stages (**Table 4**). The data at the significance level indicate that in the observation height range of 0.2−2.0 m, observation periods of 8:00−18:00, and moving speed of 0−2.0 m min^−1^, changes in H, t, and v had no significant effect on measurements. However, T, θ, and D significantly affected measurements within the observation angle range of 0−60° and planting density range of 0.2 d−d. Hence, the growing period, the angle at which the sensor was placed, and the crop planting density were the key variables influencing ultrasonic measurement. In addition, the influences of atmospheric pressure, temperature, humidity, sound wave attenuation, and moving speed within a certain range in this experiment can be neglected in the field.

**Table 4:** One-way analysis of variance (ANOVA) to investigate the effects of the growth stage (T), observation angle (θ), planting density (D), observation height (H), observation time (t), and moving speed (v) of maize and wheat on the measurement value of canopy height by ultrasonic sensor (CH_m_).

Maize	Source	Sum of squares	df	Mean square	Significant level
T	Between groups	1969070.341	4	492267.585	< 0.001 (***)
	Within groups	7335.111	10	733.511	
	Total	1976405.452	14		
θ	Between groups	1172021.991	4	293005.498	< 0.001 (***)
	Within groups	64394.062	10	6439.406	
	Total	1236416.054	14		
D	Between groups	124095.121	4	31023.780	0.012 (*)
	Within groups	54417.105	10	5441.710	
	Total	178512.226	14		
H	Between groups	8170.696	4	2042.674	0.297
	Within groups	14413.311	10	1441.331	
	Total	22584.007	14		
t	Between groups	11178.284	4	2794.571	0.376
	Within groups	23627.825	10	2362.782	
	Total	34806.109	14		
v	Between groups	15456.620	4	3864.155	0.393
	Within groups	33995.154	10	3399.515	
	Total	49451.774	14		
Wheat	Source	Sum of squares	df	Mean square	Significant level
T	Between groups	192533.594	4	48133.399	< 0.001 (***)
	Within groups	1123.690	10	112.369	
	Total	193657.284	14		
θ	Between groups	99800.448	4	24950.112	< 0.001 (***)
	Within groups	6368.467	10	636.847	
	Total	106168.916	14		
D	Between groups	58847.060	4	14711.765	< 0.001 (***)
	Within groups	4879.657	10	487.966	
	Total	63726.717	14		
H	Between groups	1396.842	4	349.211	0.261
	Within groups	2253.948	10	225.395	
	Total	3650.790	14		
t	Between groups	2464.383	4	616.096	0.436
	Within groups	5953.447	10	595.345	
	Total	8417.830	14		
v	Between groups	780.660	4	195.165	0.483
	Within groups	2090.498	10	209.050	
	Total	2871.158	14		

T is growth stage, maize growth stages include the three-leaf stage (V3), six-leaf stage (V6), nine-leaf stage (V9), twelve-leaf stage (V12), and tasseling stage (VT), and wheat growth stages include the regreening, jointing, heading, filling, and maturity stages. Treatments in rows followed by different letters differed significantly based on an F-test, and the significance of each correlation is indicated as *P< 0.05, ***P< 0.001, and NS (not significant) P > 0.05.

**Figure 6:**
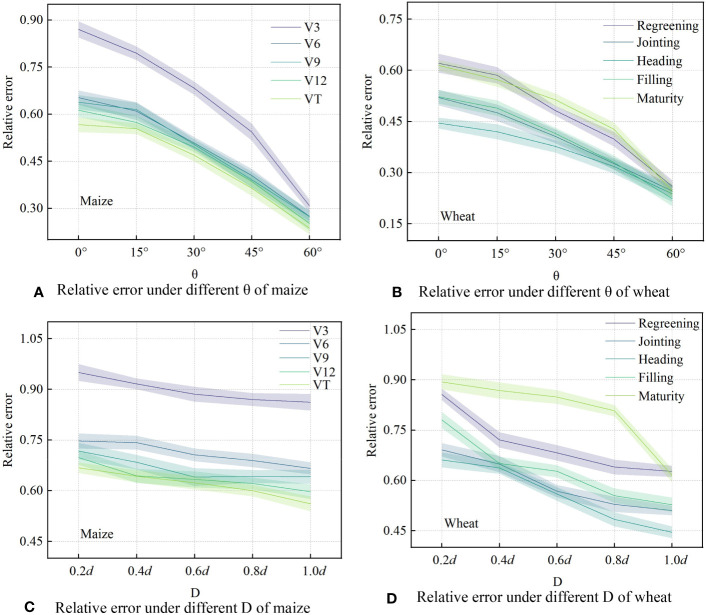
Variation in trends of relative error in the measurement value of canopy height by ultrasonic sensor (CH_m_) under different observation angles (θ) and planting densities (D) for maize **(A, C)** and wheat **(B, D)**.

**Figures 6A** and **B** show that with an increase in the observation angle, the relative errors of measurement were reduced in an orderly manner, and the downward trend became more obvious. **Figures 6A** and **C** show that the relative error for maize from V3 to V9 continued to decrease from 0.877 to 0.274. VT had the smallest relative error among the growth stages with a range of 0.246−0.568. **Figures 6B** and **D** show that the relative error of wheat in the regreening to maturity stages first decreased and then increased with a variation range of 0.223−0.621. The relative error in the heading stage was the lowest within each period, ranging from 0.238 to 0.445. This is because the canopy coverage was highest during the VT of maize and heading stage of wheat during experiment. Similarly, the measurement accuracy showed the same trend for different canopy densities (**Figures 6C, D**). As the planting density increased from 0.2d to 1.0d, the relative error range of maize decreased from 0.667−0.950 to 0.561−0.862, and that of wheat decreased from 0.661−0.894 to 0.445−0.627.

### 3.4 Establishment and assessment of the canopy height measurement calibration model

From the above analysis, it can be determined that planting density and observation angle are the main factors affecting the ultrasonic measurements. Because the measurement error from observation angle can be reduced by fixing the observation direction, it is necessary to study the measurement changes caused by crop planting density. **Figure 5** indicates that the NDVI correlates well with LAI, and this vegetation index is relatively mature and widely used in canopy measurement (Tucker, 1979). Hence, it is possible to use the NDVI to study the main factors affecting ultrasonic measurements.

The wheat leaves in the mature stage withered when the canopy height tended to be stable, and this situation did not comply with the trend that canopy height increased with increasing coverage (**Figure 4B**). In addition, crop height is stable during the late growth stages, and observing canopy height before these stages is more practical for production (Scotford and Miller, 2004; Aziz et al., 2004; Walter et al., 2019). Therefore, only the crop growth stage prior to leaf withering was studied when calibrating ultrasonic measurements to reduce the complexity of verification.

**Figure 7:**
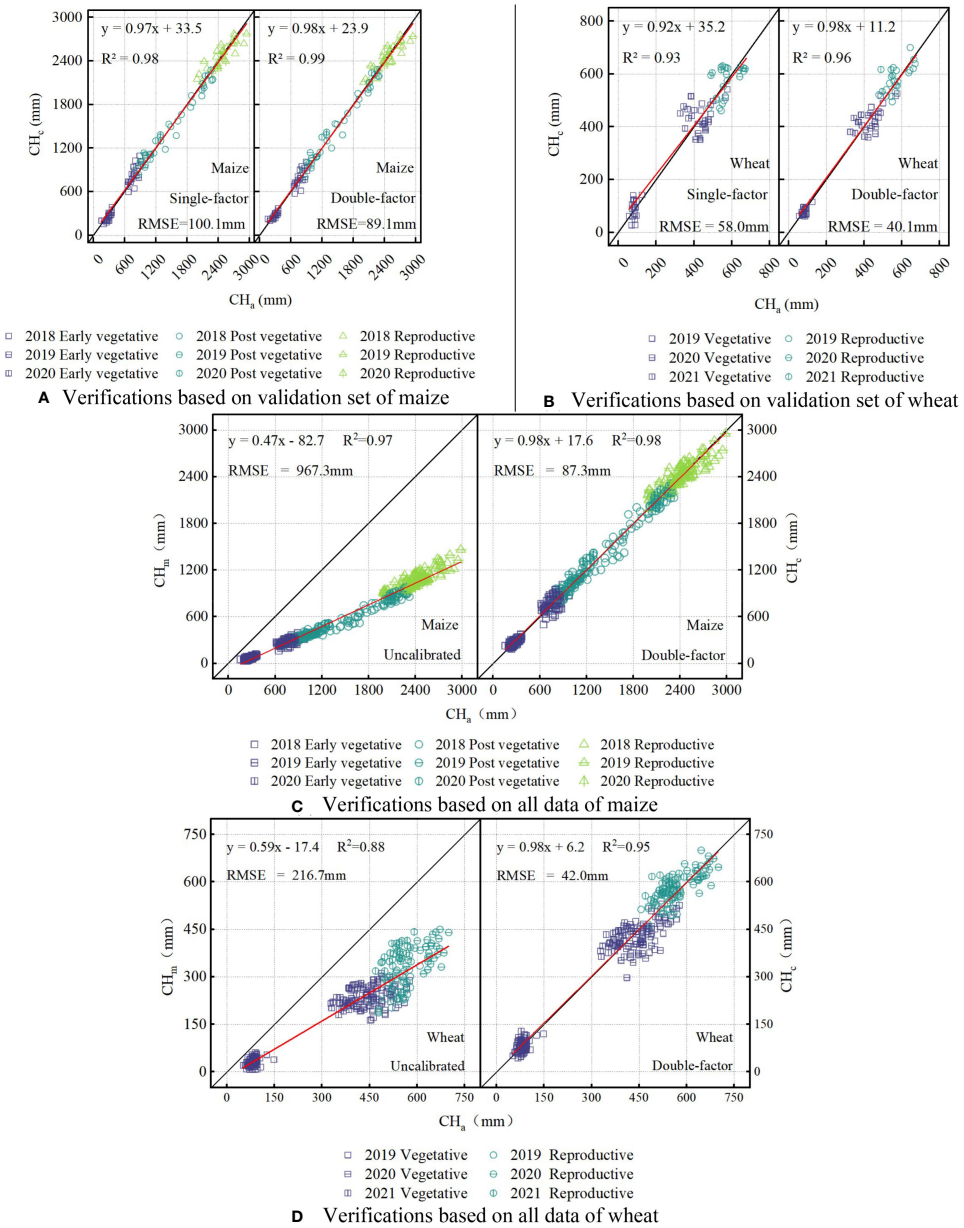
Calibrations before and after using the double-factor model based on the in-situ canopy experiment data **(A)** number of maize samples N=100. **(B)** number of wheat samples N=70. **(C)** number of maize samples N=403. **(D)** number of wheat samples N=286) from 2018 to 2021. .

Empirical calibration models were constructed using all data from the in-situ canopy experiments. These data were divided into training set and validation set by random sampling method (Shao et al., 2022; Shao et al., 2023; Zhang et al., 2023). Taking the four-year data as a whole when dividing these sets can enrich the year-differences of data set, and this method of data processing can improve the adaptability of the model to different years and make the model more robust. Stratified random sampling method (Zhu et al., 2020) was used in this study and all data from each growth stage was randomly divided into training and validation sets in a ratio of approximately 3 to 1. In all data, there were 403 groups of maize data in total (303 groups in the training set and 100 groups in validation set), and 286 groups of wheat data in total (216 groups in the training set and 70 groups in validation set). The statistical characteristics of the sample data are shown in **Supplementary Table. S1**. The single-factor and double-factor calibration models were constructed based on above data and the method of multiple non-linear regression (Berthold and Hand, 2006), and the output was CH_c_. In the single-factor model, only CH_m_ was considered as input. For the double-factor model, the input parameters were CH_m_ and NDVI. In this study, both NDVI and CHm (R^2^ of 0.64−0.71 between them) increased with crop growth, but due to the growth difference between years and the saturation effect (the plant height was still increasing when the canopy coverage reached its limit) of NDVI, the variance inflation factor of maize and wheat (the VIF of 2.78−3.45) were within an acceptable range. Therefore, it is reasonable to build the model based on the above research. When establishing the calibration model, the equations (**Table 5**) were first obtained based on the training set, and then the model were evaluated based on the validation set (**Figures 7A, B**). For both maize and wheat, the R^2^ value of the double-factor model was consistently higher than that of the single-factor model. For example, the determination coefficient of the linear fitting equation in the model evaluation results increased from 0.986 to 0.989 for maize, and from 0.925 to 0.964 for wheat when using double-factor models. Meanwhile, there were decreases in the RMSE (100.1 mm to 89.1 mm) for maize and the RMSE (58.0 mm to 40.1 mm) for wheat.

**Table 5:** Nonlinear regression models based on the in-situ canopy experiment data (maize N=303, wheat N=216).

Crop	Input factor	Calibration model formula	
Maize	CH_m_(*x*_1_)	CH_c_=46.548 + 2.944*x*_1_–0.000669*x*_1_^2^	R^2^ = 0.987
	CH_m_(*x*_1_), NDVI(*x*_2_)	CH_c_=118.07 + 2.321*x*_1_–183.474*x*_2–_0.000657*x*_1_^2^_ + _201.804*x*_2_^2^ +0.685*x*_1_ *x*_2_	R^2^ = 0.989
Wheat	CH_m_(*x*_1_)	CH_c_=6.76 + 2.654*x*_1_–0.002767*x*_1_^2^	R^2^ = 0.927
	CH_m_(*x*_1_), NDVI(*x*_2_)	CH_c_=–99.398 + 0.806*x*_1 + _274.845*x*_2_–0.000579*x*_1_^2^_ + _463.433*x*_2_^2^ –0.028*x*_1_ *x*_2_	R^2 ^= 0.961

The output parameter of the model is the canopy height calibration value (CH_c_), and the input factors include the canopy height measurement value by ultrasonic sensor (CH_m_) and the vegetation normalization index (NDVI) at the same observation location.

Then, CH_m_ was calibrated based on all data from the in-situ canopy experiments (maize N=403, wheat N=286), and the results before and after using the double-factor model are shown in **Figures 7C** and **D**. The accuracy of ultrasonic measurements was significantly improved by using the double-factor calibration model in **Table 5**. The measurement accuracy RMSE of maize decreased from 967.3 mm to 87.3 mm, and the RMSE of wheat decreased from 216.7 mm to 42.0 mm.

### 3.5 Accuracy evaluation of calibration model within different years

**Figure 8:**
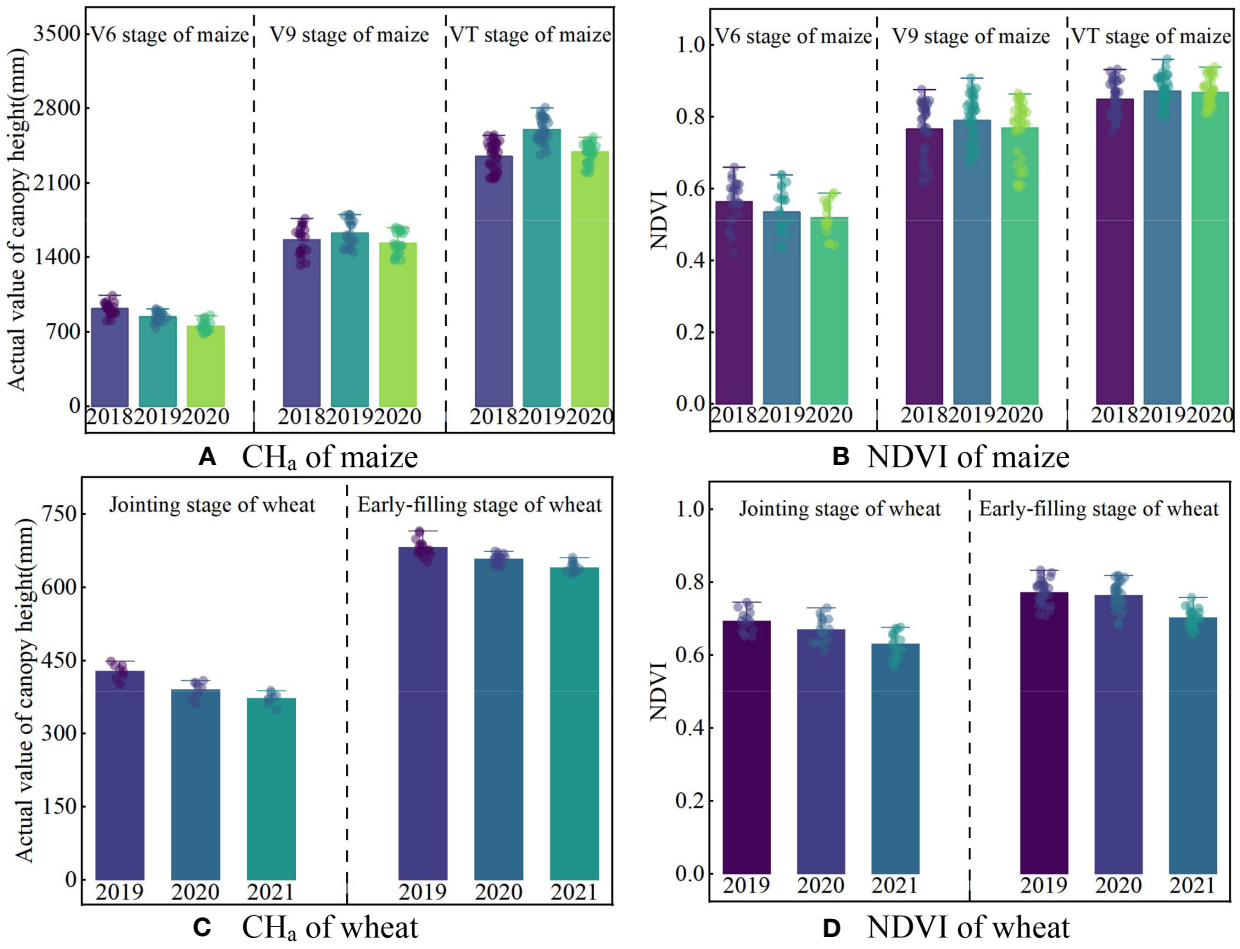
Actual values of canopy height and NDVI under each water treatment during typical crop growth stages of maize **(A, B)** and wheat **(C, D)**.

The actual value of canopy height and NDVI of maize (V6, V9, and VT) and wheat (jointing and pre-filling) are shown in **Figure 8**. Maize canopy height increased from 735−1042 mm to 2134−2810 mm during the V6−VT period. The NDVI of maize increased from 0.42−0.66 in V6 stage to 0.76−0.92 in VT stage as leaves grew. The CH_a_ of wheat was lower than that of maize, and increased from 298−449 mm in the jointing stage to 533−716 mm in the early-filling stage. The CH_a_ and NDVI fluctuations observed during the early-filling stage were similar to those observed during the jointing stage.

**Figure 9:**
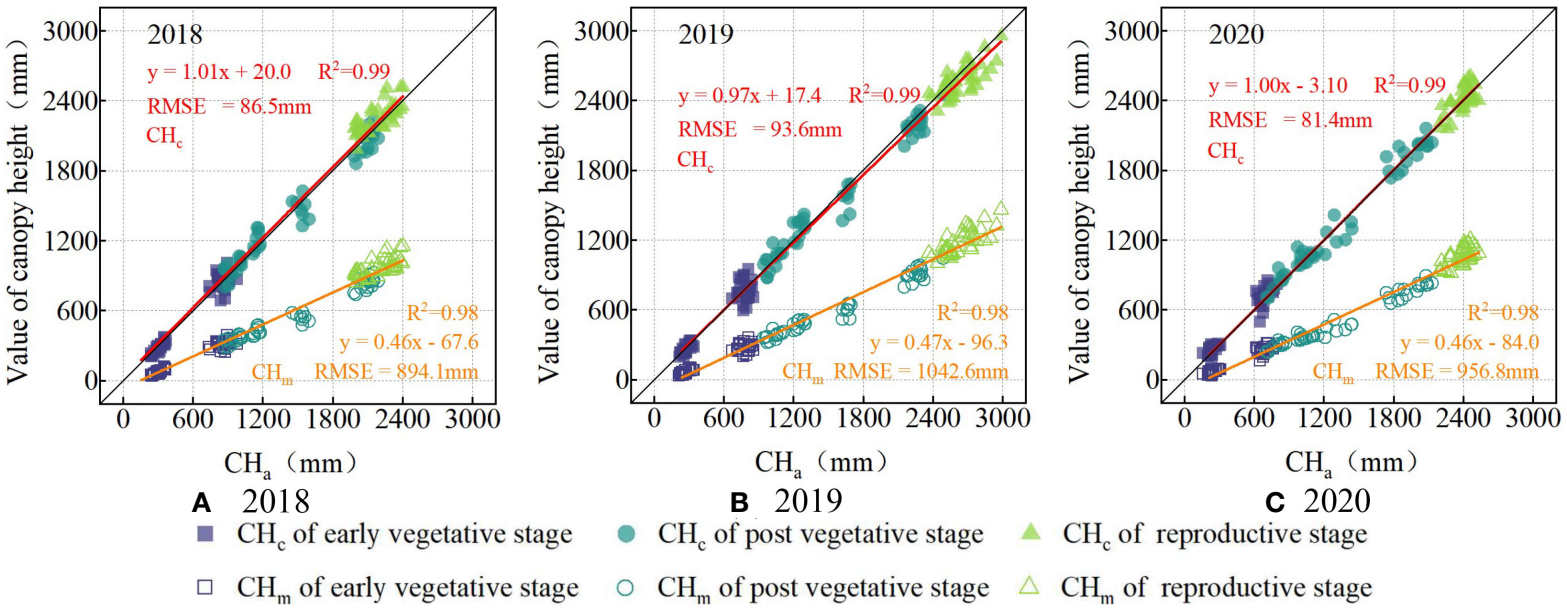
The scatter-fit plot before and after calibration of maize in 2018 **(A)**, 2019 **(B)**, and 2020 **(C)**. CH_c_ (in red) is the calibrated value of canopy height by calibration model, and CH_m_ (in yellow) is the measured value of the canopy height by ultrasonic sensor.

**Figure 10:**
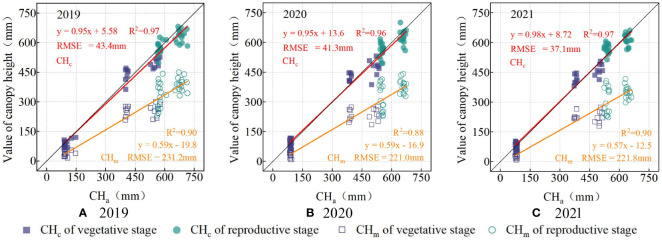
The scatter-fit plot before and after calibration of wheat in 2019 **(A)**, 2020 **(B)**, and 2021 **(C)**. CH_c_ (in red) is the calibrated value of canopy height by calibration model, and CH_m_ (in yellow) is the measured value of the canopy height by ultrasonic sensor.

The double-factor model (**Table 5**) output for each year based on the experiment data (**Table 3**) is shown in **Figure 9**. The RMSE values for maize (**Figure 9**) was diminished from a range of 894.1−1042.6 mm to 81.4−93.6 mm after calibration. Similarly, the RMSE for wheat (**Figure 10**) decreased from 221.0−231.2 mm to 37.1−43.4 mm. There was no obvious difference in calibration effects between different years.

**Figure 11:**
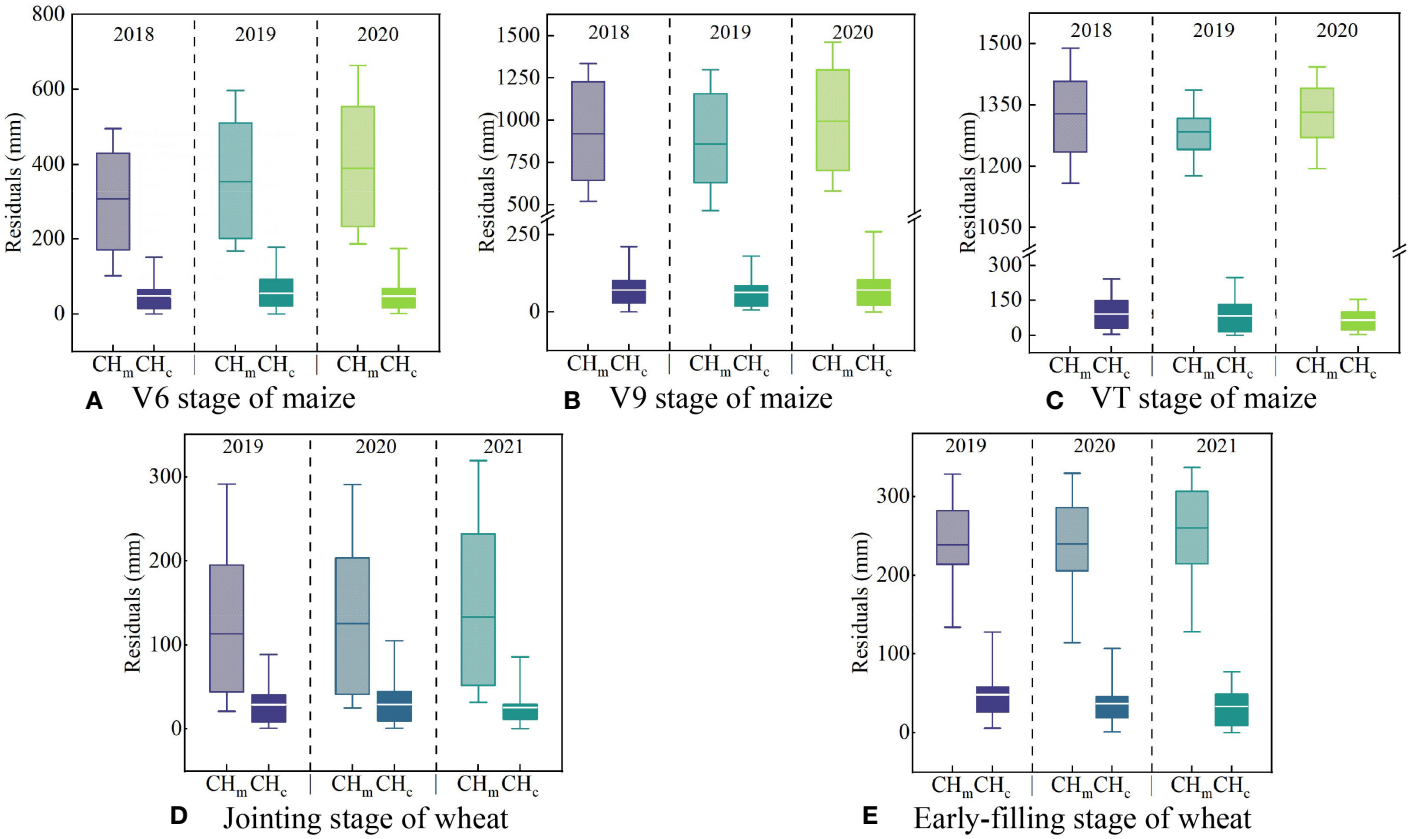
The residual box plots before and after calibration during typical crop growth stages of maize **(A–C)** and wheat **(D, E)**. CH_c_ is the calibrated value of canopy height by calibration model, and CH_m_ is the measured value of the canopy height by ultrasonic sensor.

**Figure 11** displays the residuals before and after calibration of canopy height measurements during growth stages of maize (V6, V9, and VT stages) and wheat (jointing and early-filling stage). The residual of CH_m_ was smaller in higher canopy coverage. Considering the jointing stage of wheat as an example (**Figure 11D**), the mean residuals of CH_m_ were 2019 (112 mm)<2020 (125 mm)<2021 (132 mm). Furthermore, maize (102−1491 mm) exhibits a greater CH_m_ residual than wheat (21−372 mm), owing to its lower planting density and higher overall canopy height. After the double-factor model calibration, the mean CH_a_ residuals of maize decreased from 307−1334 mm to 47−91 mm, the mean CH_a_ residuals of wheat decreased from 113−268 mm to 26−49 mm, and there was no obvious difference in the calibration results in different years.

## 4 Discussion

### 4.1 The parameters influencing ultrasonic measurements and research innovations

The ultrasonic sensor is a device with good stability and repeatability in distance measurement, but it has relatively low precision in measuring maize or wheat canopy height. The experiment results show that the observation height (0.2-2.0 m), observation period (8:00-18:00) and relative moving velocity (0-2.0 m min^-1^) have no significant effect on measurement results (P≥0.05). It can be seen that the effect of distance change on canopy height is negligible in real conditions, although this will cause a change in the observed area, and the sound attenuation increases with the increase of propagation distance. At the same time, although ambient temperature changes can significantly affect ultrasonic propagation (Scotford and Miller, 2004), the built-in temperature compensation module (-40−70 °C) guarantees that temperature changes (16-38 °C) during the experiment will not interfere with the ultrasonic sensor measurements. Barmeier et al. (2016) found that relative moving velocity affects ultrasound measurements, which is contrary to the conclusions of this study. This is due to the fact that the center pivot system cannot move at a high speed (at a low speed of 0-2.0 m min^-1^) and the flat pavement in the experiment can effectively avoid the vibrations and oscillations of the ultrasonic system. Of course, it is possible to try to include stabilization devices in subsequent studies based on this conclusion. In addition, the results showed that the growth period (V3-R of maize, green—pre-filling stage of wheat), observation angle (0-60°), and planting density (0.2-1.0 times of the standard planting density) had significant effects on ultrasound measurement (P<0.05). Combined with **Figures 4** and **5**, the above results can be attributed to the change in canopy coverage within the sensor's field of view. Studies have shown that only a plane with sufficient area at a suitable angle can effectively reflect the ultrasonic signal (McKerrow and Harper, 2002; Andújar et al., 2012). In this experiment, maize plants were widely spaced, wheat leaves were narrow, and their leaves grow obliquely. As a result, at low leaf densities, the effective signal of the sound wave was reflected by the lower canopy leaves or soil, resulting in measurements lower than the actual canopy height. This rationale explains why Sui and Baggard (2018) and Chang et al. (2017) obtained relatively accurate measurements for soybean (the RMSE of 58 mm), cotton (the RMSE of 31 mm), and blueberry (the RMSE of 23 mm). The leaves of these crops are more horizontally oriented and denser than those of maize and wheat. This orientation and density effectively reflect acoustic signals, as noted by Yuan et al. (2018).

Some scholars (Aziz et al., 2004; Farooque, 2013; Chang et al., 2017) have measured the performance of ultrasonic sensors in the laboratory or with the crops in a single growth period, and some influencing factors in their research have been measured and analyzed. However, few studies have identified the main factors influencing ultrasound measurements in the field environment through continuous and systematic multi-year maize and wheat experiments, as in the present study. At present, most of the crop field research on ultrasonic sensors is mainly focused on the direct application without calibration in field. For example, Sui and Baggard (2018) integrated a variety of sensors (including ultrasonic sensors and infrared temperature sensors) into a center pivot system to measure the soybean canopy, but their experiment did not consider using spectral reflectance data to further assist the ultrasonic sensor in obtaining more accurate measurements. Of course, the reason why they did not conduct such a study may also be related to the fact that the experimental crop was soybean, but this is more indicative of the importance of the research on maize and wheat in this study. Therefore, based on their meaningful and valuable research foundation, this study attempts to combine NDVI and ultrasonic data to accurately measure the canopy height of maize and wheat. Similarly, Pittman et al. (2015) built a tractor-based platform with an ultrasonic sensor, the NDVI sensor, and the laser sensor, but their model of alfalfa canopy height was constructed using only ultrasonic and laser data. In addition, many scholars have obtained more accurate ultrasonic measurements by continuously and repeatedly measuring the same experimental plot (Scotford and Miller, 2004) or taking the 95.5-100th percentile (Jimenez-Berni et al., 2018; Madec et al., 2017; Sun and Li, 2016), but these operations will affect the measurement efficiency or the integrity of the original data. In summary, the content of this study can serve as a further supplement and improvement in their research. In this study, we attempted to introduce the canopy coverage into the calibration model. It is possible for the methods of fitting with empirical formulas (Section 3.4) to compensate for the errors caused by ultrasound. The calibration model achieved satisfactory results in the measurement of canopy height in maize and wheat, and the calibration results for different years and growth periods had satisfactory accuracy (Section 3.5), which shows the reliability and applicability of this model.

Moreover, it is necessary to analyze the propagation and reflection mechanism of ultrasonic signals under complex canopy conditions to improve the adaptability of the model to different regions and crops. Therefore, it is important to use more advanced image or laser point cloud techniques to understand the distribution of the leaves, and to optimize the measurement results in combination with more accurate mathematical models. In addition, due to the rapid technological innovation, there are other methods of measuring canopy height in addition to ultrasonic technology, such as LiDAR or UAS imagery technology. Therefore, in the following section, a comparative analysis of various measurement methods is presented to further clarify the characteristics of the calibrated ultrasonic measurement method.

### 4.2 Comparison of ultrasonic with other canopy height measurement techniques

In addition to ultrasonic measurements, the technologies widely used in canopy height measurement include LiDAR (Jimenez-Berni et al., 2018; Walter et al., 2019) and UAS imagery (Friedli et al., 2016; Madec et al., 2017). Among them, LiDAR is based on the principle of time of fly (Sun et al., 2017). The canopy height was obtained by converting the raw data from LiDAR into a three-dimensional (3D) point cloud. This technique has achieved good measurement accuracy in various researches, and for example, the measurement accuracy of rye (Busemeyer et al., 2013), barley (Tilly et al., 2015), rice (Tilly et al., 2014), cotton (Sun and Paterson, 2017), and pea (Underwood et al., 2017) is 24 mm, 30−60 mm, 50 mm, 35−65 mm, and 46 mm respectively. UAS Imagery uses a UAS platform with a high−resolution camera. This technique retrieves a digital surface model (3D point cloud) of the canopy from multiple photographs using the triangulation principle (Yuan et al., 2018) and estimates plant height based on this principle. This method is also widely used in the measurement of barley (Bendig et al., 2014), sorghum (Malambo et al., 2018), shrub (Díaz-Varela et al., 2015; Fraser et al., 2016) and forest (Dandois and Ellis, 2013; Puliti et al., 2015) canopy height, and the measurement accuracy of UAS imagery on above canopy types is 100, 120−240, 80−200, and 400−1400 mm respectively.

After conducting a comparison, researchers (Yuan et al., 2018; Yuan, 2019) found that the ultrasonic measurements had the lowest accuracy, the LiDAR measurements had the highest accuracy (Madec et al., 2017), and the UAS imagery measurements had the moderate accuracy. This is because LiDAR has the high resolution (3−5 mm) and the strong penetration (Madec et al., 2017). LiDAR can directly generate a fine canopy 3D point cloud (Friedli et al., 2016) from the raw measurements obtained via top-down scanning (Walter et al., 2019). The UAS imagery indirectly generates 3D point clouds using a large number of overlapping canopy images at different angles. UAS requires a high flight altitude to increase the measurement range and prevent propeller airflow from disturbing the canopy. Due to the limited penetration ability of UAS imagery when photographing canopy (Yuan et al., 2018), this technique cannot accurately identify canopy features such as wheat ears or corn male ears. Some scholars (Grenzdörffer, 2014; Bareth et al., 2016; Van der Voort, 2016) have got the similar conclusion that UAS lacks effective canopy top reconstruction capacity. As a result, the accuracy of UAS imagery was lower than that of lidar data. Compared to the above two methods, ultrasonic sensors are affected by the large deviation in the direction of reflection of leaves when using ultrasonic measurements (Yuan et al., 2018) although they have the resolution of 0.172 mm. The top of the canopy of maize or wheat cannot form a continuous plane. Therefore, the error in the ultrasonic measurement value is relatively large.

In this study, it was verified that the calibration model reached the RMSE (ultrasonic measurement error) of 87.3 mm for maize and 42.0 mm for wheat. Other studies have also taken measurements of the same crop canopy. The measurement accuracy of LiDAR for maize is 50−170 mm (Li et al., 2015; Hu et al., 2023: Anthony et al., 2014) and 17−50 mm for wheat is (Virlet et al., 2016; Jimenez-Berni et al., 2018; Yuan et al., 2018). The measurement accuracy of UAS imagery for maize is 90−190 mm (Varela et al., 2017; Malambo et al., 2018) and 30−90 mm for wheat (Holman et al., 2016; Madec et al., 2017; Yuan et al., 2018). The calibrated ultrasonic measurement accuracy was already at the same level as the measurement accuracy of the above technique after comparing the above results. Considering that lidar is expensive (Fricke and Wachendorf, 2013), and UAS has limited payload and flight time (Deery et al., 2014). In addition, the processing of large amounts of point cloud data is cumbersome (Singh et al., 2016), and special software is required for accurate information evaluation (Gebbers et al., 2011; Llorens et al., 2011). In contrast, ultrasonic sensors are a relatively low−cost (Sui et al., 2012) and user−friendly (Madec et al., 2017). The ultrasonic output is easy to handle and its relatively low cost allows users to use multiple sensors in parallel (Andújar et al., 2012). The installation on the platform of ground−based agricultural machinery, allows the sensing equipment to remain on site, and enables quick and rapid canopy measurements. These systems facilitate the timely implementation of key management decisions. Hence, the established calibration models in this study have effective application values.

### 4.3 Limitations and suggestions

A four-year field experiment of the ultrasonic sensor was conducted based on of the field data in this study. The constructed ultrasonic calibration model was verified as suitable for canopy height measurements (maize/wheat) in different years and the constructed system can acquire CHm and canopy NDVI data stably and efficiently based on a center pivot. Although the use of spectral sensors inevitably increases the cost, the price of this type of sensor combination is still lower than that of LiDAR. Additional observational indicators such as spectral data indicate that more comprehensive and real-time crop growth information can be obtained. These indicators can help ultrasonic measurement systems to play a more important role in the field of non−destructive prediction of important crop growth parameters such as leaf area index and aboveground biomass (Scotford and Miller, 2004; Fricke and Wachendorf, 2013; Pittman et al., 2015). Compared with the UAS, the ultrasonic measurement system built by the ground-based platform does not have an advantage in the data throughput of canopy information acquisition (Yuan et al., 2018). However, this disadvantage can be compensated by the ease with ultrasonic data. The relatively slow measurement speed allows for sufficient data processing time for platforms such as center pivot irrigation machines. These data processing time make it possible for the system to make flexible and timely management decisions. In follow-up research, researchers can try to efficiently combine the measurement system with the agricultural machinery platform toward the field management goal of “canopy measurement−data processing−decision implementation”.

Future research should focus on improving canopy height measurements throughout the growth cycle of most field crops. Cost comparison tests can be carried out under field conditions. These tests help to determine the types of sensors that are suitable for long-term monitoring. In addition, it is important to conduct the research on the optimal configuration of multi-type sensor combinations. These tests can help to improve the observation efficiency of multi-sensor systems.

It is undeniable that LiDAR and UAS Imagery possess numerous irreplaceable measurement advantages. The use of fine imaging or 3D cloud mapping technology facilitates further analysis of the reflection propagation principle of ultrasonic signals in complex crop canopy structures. Therefore, combining ultrasonic systems with various remote sensing technologies (e.g., LiDAR, UAS, and satellites) to complement the advantages of multi-source data is another important research direction (Schirrmann et al., 2017).

## 5 Conclusion

In this study, the CH_m_ values of maize and wheat were obtained using a self-built ultrasonic ranging system. In addition to maintaining good measurement stability and repeatability, this system has proven to be a long-term field measurement device. Moreover, combined with the canopy height measurement experiment, the canopy coverage significantly affected the ultrasonic measurement results (P< 0.05). Under the conditions of changing the observation height within 0.2−2.0 m, the observation period within 8:00−18:00, and the relative moving speed within 0−2.0 m min^−1^, the canopy height measurements were not significantly affected (P > 0.05).

Furthermore, the canopy coverage represented by the NDVI is an important parameter for constructing nonlinear regression models. With this parameter, these empirical models can be quite useful for ultrasonic measurements of canopy height within different years. After calibration, the measurement accuracy RMSE decreased from 967.3 mm to 87.3 mm for maize canopy height, and the RMSE decreased from 216.7 mm to 42.0 mm for wheat. The results of this study can be applied to large-scale agricultural machinery platforms and can provide technical support for real-time field management.

## Data availability statement

The original contributions presented in the study are included in the article/**Supplementary Material**. Further inquiries can be directed to the corresponding author.

## Author contributions

YZ: Conceptualization, Data curation, Formal analysis, Investigation, Methodology, Project administration, Software, Validation, Visualization, Writing – original draft. XH: Conceptualization, Methodology, Supervision, Writing – review & editing. DC: Supervision, Writing – review & editing, Methodology. MS: Methodology, Supervision, Writing – review & editing. YW: Data curation, Methodology, Supervision, Writing – review & editing. ZW: Data curation, Investigation, Project administration, Writing – review & editing. FM: Writing – review & editing. HY: Conceptualization, Funding acquisition, Resources, Supervision, Writing – review & editing.
