# Supplementary material for: Calibrating ultrasonic sensor measurements of crop canopy heights: a case study of maize and wheat
Source: Front Plant Sci. 2024 Jun 5;15:1354359. doi: 10.3389/fpls.2024.1354359 (PMC11188359; doi:10.3389/fpls.2024.1354359)
Supplement: Supplementary file 1 [file DataSheet_1.docx]

**Table. S1. Statistical characteristics of sample data.**

| Crop | Data set | Parameter | Maximum  value | Minimum  value | Average  value | Standard deviation | Coefficient of variation |
| --- | --- | --- | --- | --- | --- | --- | --- |
| Maize | Training | CH_a_ (mm) | 2984 | 198 | 1433 | 834 | 58% |
|  |  | CH_m_ (mm) | 1462 | 33 | 583 | 391 | 67% |
|  |  | NDVI | 0.926 | 0.002 | 0.632 | 0.267 | 42% |
|  | Validation | CH_a_ (mm) | 2947 | 155 | 1475 | 852 | 58% |
|  |  | CH_m_ (mm) | 1326 | 38 | 605 | 402 | 66% |
|  |  | NDVI | 0.897 | 0.002 | 0.634 | 0.260 | 41% |
| Wheat | Training | CH_a_ (mm) | 716 | 51 | 422 | 216 | 51% |
|  |  | CH_m_ (mm) | 450 | 7 | 230 | 133 | 58% |
|  |  | NDVI | 0.843 | 0.297 | 0.642 | 0.175 | 27% |
|  | Validation | CH_a_ (mm) | 677 | 57 | 425 | 213 | 50% |
|  |  | CH_m_ (mm) | 414 | 8 | 231 | 130 | 56% |
|  |  | NDVI | 0.858 | 0.310 | 0.645 | 0.177 | 27% |

**NOTE: CH_a_ is the actual value of canopy height, CH_m_ is the canopy height meas****urement value by ultrasonic sensor, and NDVI is the vegetation normalization index.**

| 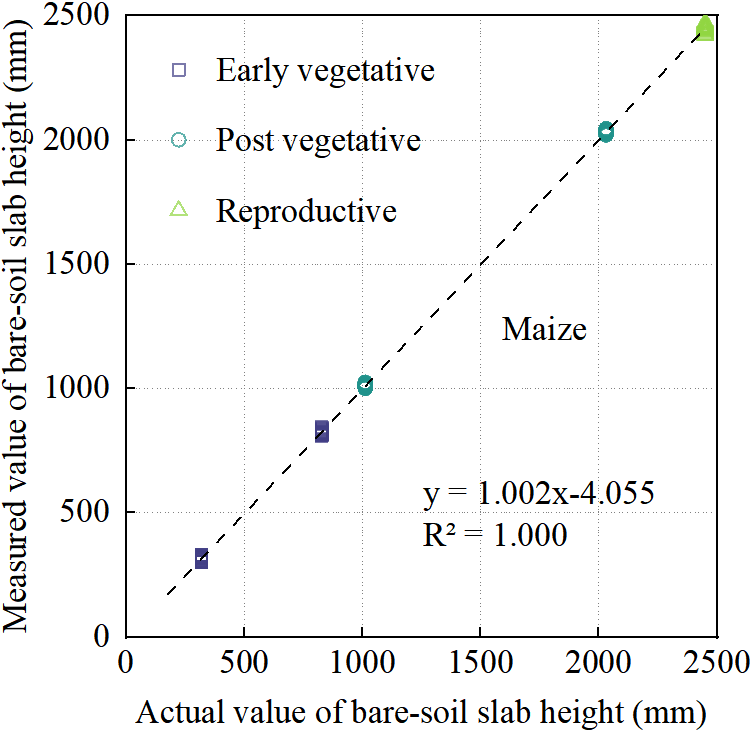 | 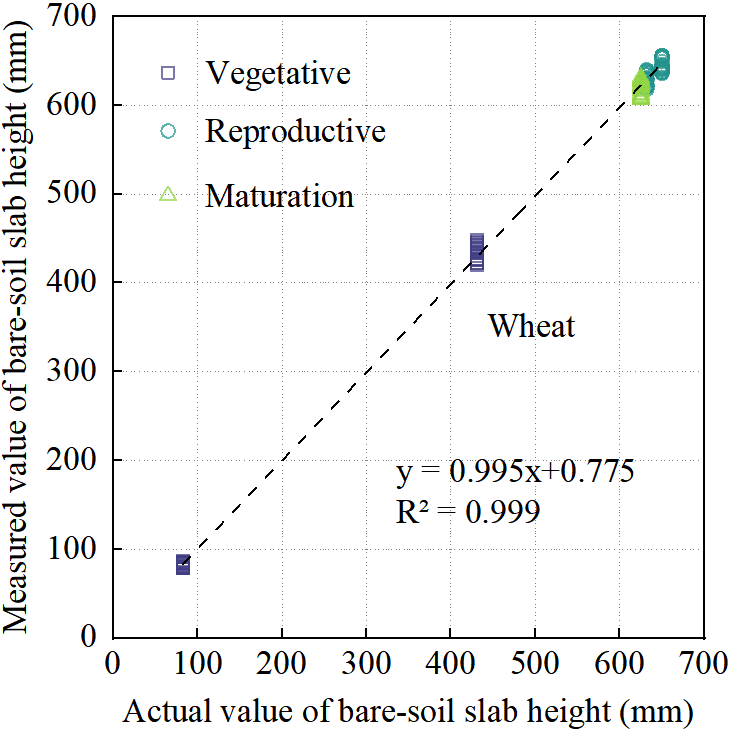 |
| --- | --- |
| (a) Under maize observations conditions | (b) Under wheat observations conditions |

**Fig. S1. Verification of the measurement reliability of ultrasonic sensor based on the bare-soil board at each critical growth stage of crop. NOTE: The dashed lines indicate the best fit line.**

| 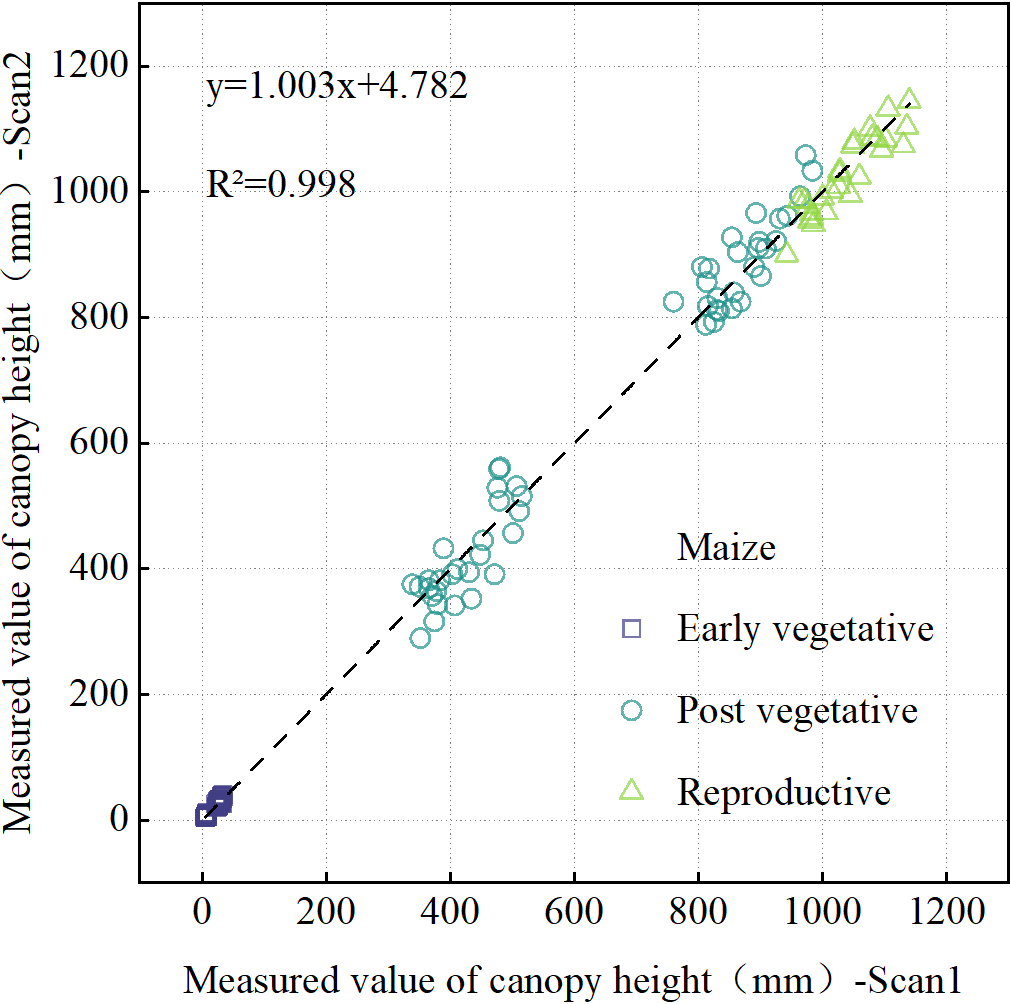  (a) Observations in same direction of maize | 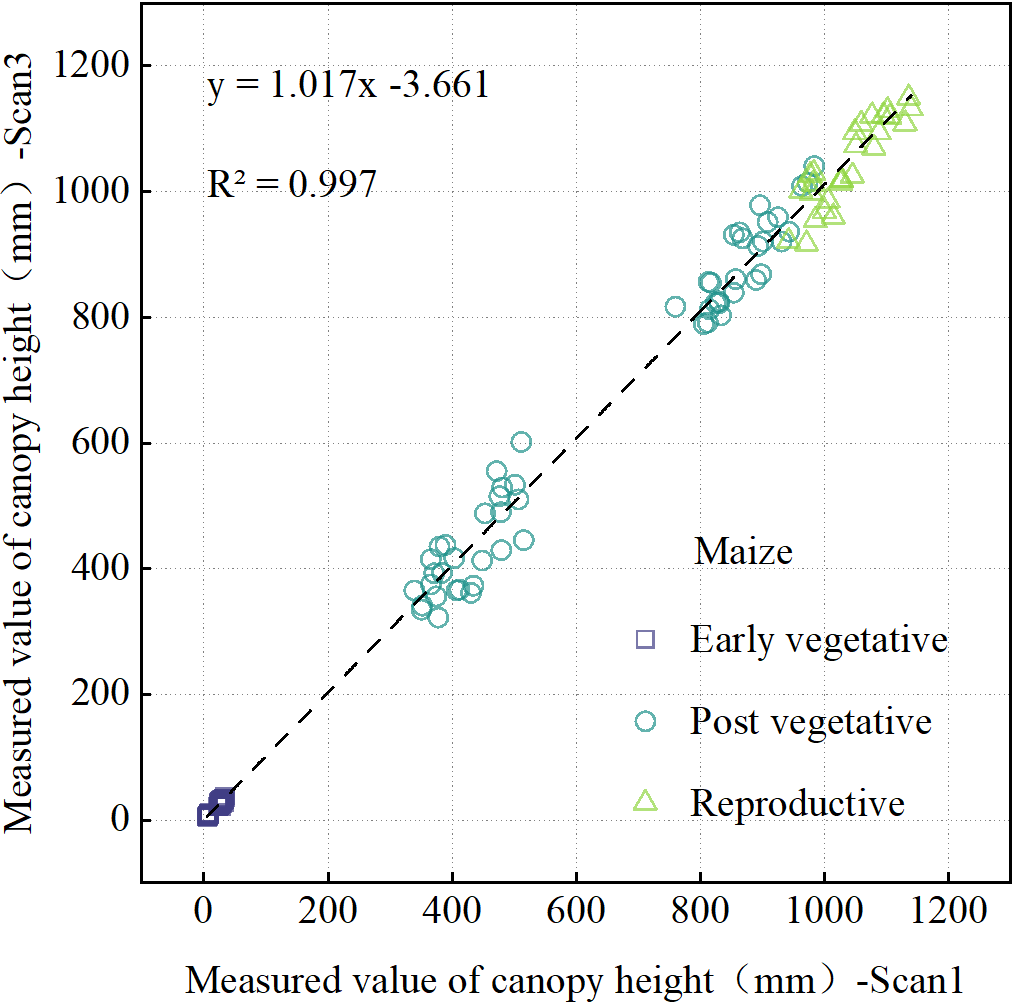  (b) Observations in opposite direction of wheat |
| --- | --- |
| 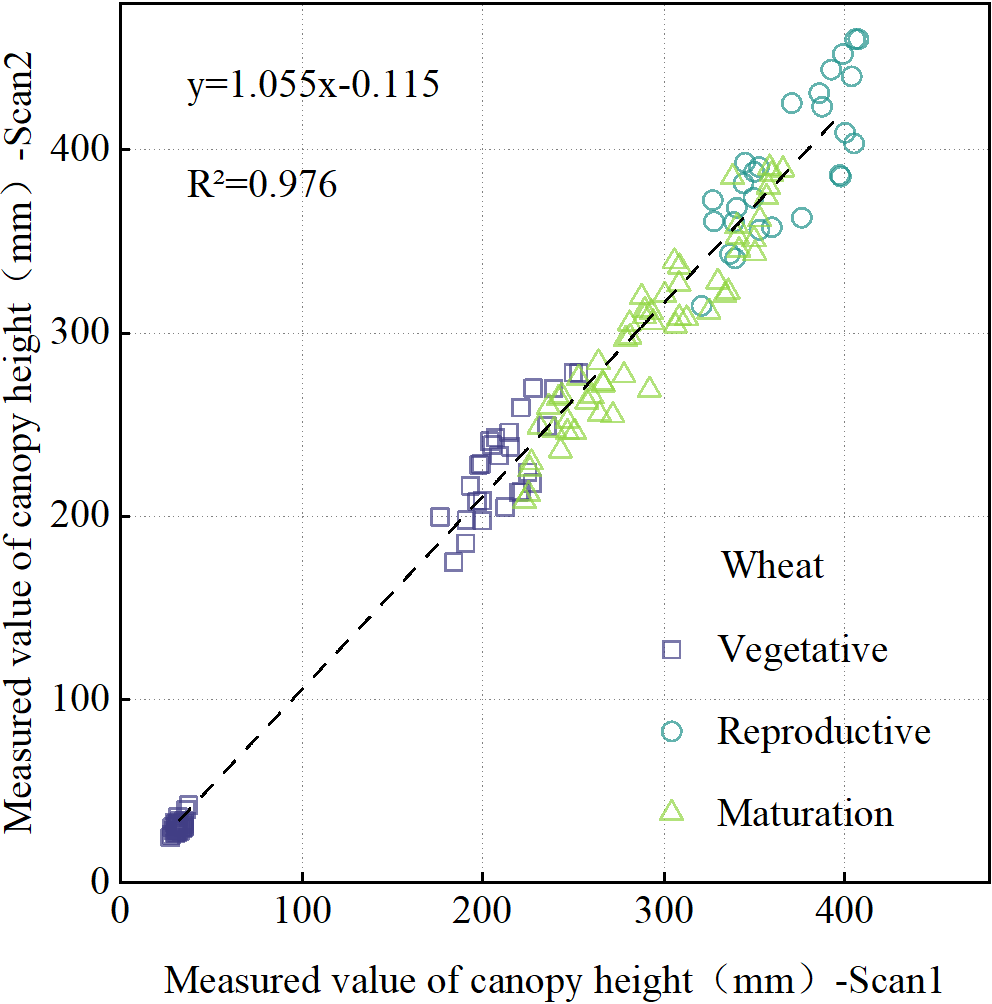  (c) Observations in same direction of wheat | 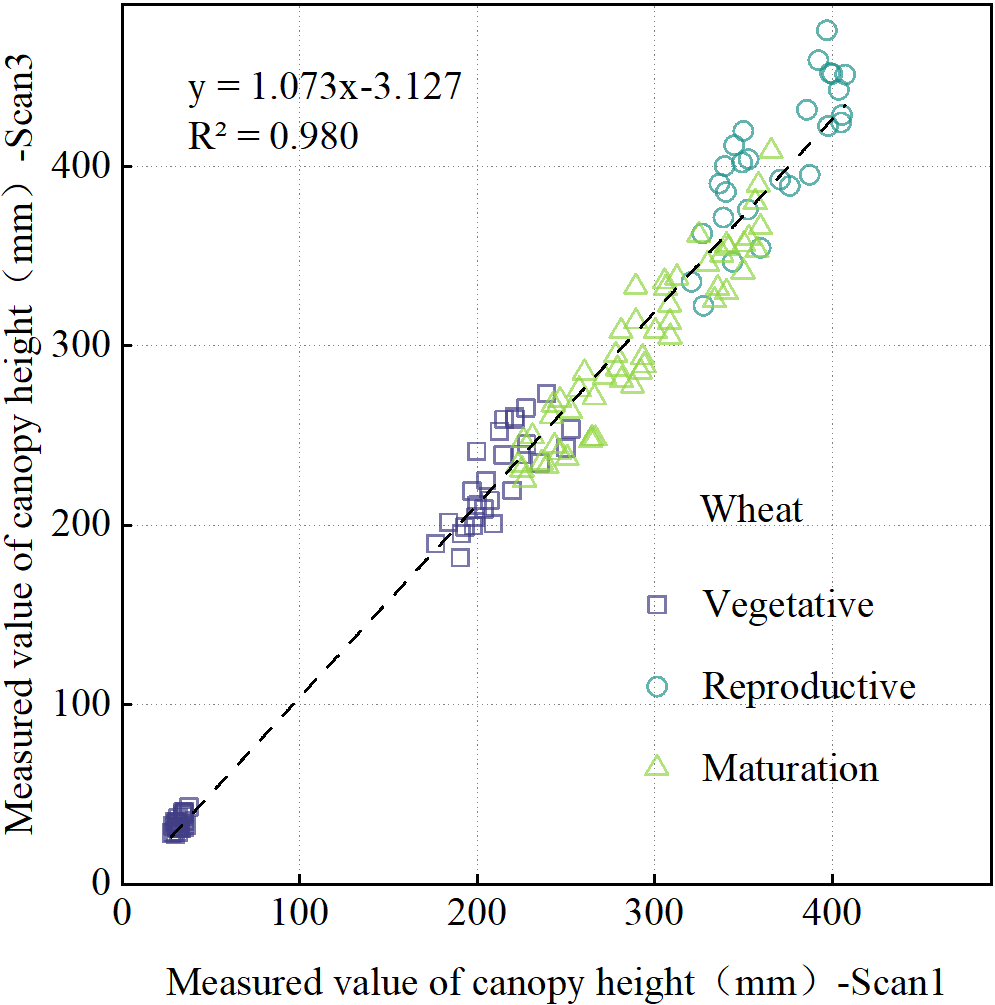  (d) Observations in opposite direction of wheat |

**Fig. S2. Verification of the measurement reliability of ultrasonic sensor based on crop canopy at each critical growth stage of crop. NOTE: The dashed lines indicate the best fit line.**
